# Associations between cardiac function and retinal microvascular geometry among Chinese adults

**DOI:** 10.1038/s41598-020-71385-0

**Published:** 2020-09-09

**Authors:** Lihua Huang, Wei-Qing Chen, Izzuddin M. Aris, Louis L. Y. Teo, Tien Yin Wong, Angela S. Koh, Ling-Jun Li

**Affiliations:** 1 Department of Medical Statistics and Epidemiology, Guangzhou Key Laboratory of Environmental Pollution and Health Assessment, Guangdong Provincial Key Laboratory of Food, Nutrition and Health, School of Public Health, Sun Yat-Sen University, Guangzhou, 510080 China; 2 Division of Chronic Disease Research Across the Lifecourse, Department of Population Medicine, Harvard Medical School and Harvard Pilgrim Health Care Institute, Boston, MA 02215 USA; 3 National Heart Centre Singapore, 5 Hospital Drive, Singapore, 169609 Singapore; 4 Duke-NUS Medical School, Singapore, 169857 Singapore; 5 Division of Obstetrics and Gynecology, KK Women’s and Children’s Hospital, Singapore, 229899 Singapore; 6 Obstetrics and Gynecology Academic Clinical Program, Duke-NUS Medical School, Singapore, 169857 Singapore; 7 Singapore Eye Research Institute, Singapore National Eye Centre, The Academia, 20 College Road, Discovery Tower level 6, Singapore, 169856 Singapore; 8 Department of Obstetrics and Gynecology, Yong Loo Lin School of Medicine, 1E Kent Ridge Road, Level 12, Singapore, 119228 Singapore

**Keywords:** Cardiology, Diseases

## Abstract

Abnormal retinal microvascular geometry has been associated with cardiac remodeling and heart failure. However, its relation to cardiac function, prior to clinical disease has not been explored. In this cross-sectional study, 50 participants (mean age 62.5 ± 11.7 years) without cardiovascular disease (CVD) were recruited from the Cardiac Ageing Study. Transthoracic echocardiography imaging was performed to measure cardiac function indices, and retinal imaging was used to measure retinal vascular caliber and retinal vascular geometric indices. Multiple linear regressions were applied to examine associations between indices of cardiac function and retinal microvasculature, adjusting for age, sex, body mass index, mean blood pressure and comorbidity (i.e. hypertension, diabetes and dyslipidemia). After adjusting for confounders, each unit decrease in peak systolic septal mitral annular velocity (Septal S′) indicating poorer left function was associated with smaller retinal venular branching angle (β: − 2.69°; 95% CI − 4.92, − 0.46). Furthermore, each unit increase in peak velocity flow in late diastole by atrial contraction (MV A Peak) indicating poorer left atrial function was associated with lower retinal venular fractal dimension (− 0.13Df; − 0.25, − 0.004). Our findings suggested a relationship between poorer cardiac function and suboptimal retinal microvascular geometry, among Chinese without CVD.

## Introduction

The retina is a site where microcirculation can be viewed directly. The technique of retinal imaging has provided a unique opportunity to study the morphology of human microcirculation in vivo in an accurate, non-invasive manner^1^. Recent cross-sectional studies suggested that subclinical cardiac remodelling among patients without documented cardiovascular disease (CVD) was associated with retinal arteriolar narrowing and/or venular widening^2,3^, raising the possibility that adverse microvascular alteration may underlie the subclinical stage of CVD and even asymptomatic heart failure (HF)^4^. However, whether such retinal microvascular abnormality is coupled with subclinical alteration in cardiac function other than cardiac structure remains understudied. Therefore, we conducted this study to explore the associations between cardiac function and retinal microvascular morphology including novel geometric indices in a community-based population without CVD. We hypothesized that subclinical alteration in cardiac function might be associated with suboptimal retinal microvascular geometry.

## Results

The mean (SD) age and male count (%) in our study were 62.54 (11.74) years and 27 (54%), respectively. The majority of participants had reported history of hypertension and dyslipidaemia (Table 1). Compared with patients with E/A ratio > 1.0, those with E/A ratio ≤ 1.0 were older, more likely to have combination of co-morbidities and with larger waist-to-hip ratio, higher SBP and lower retinal venular fractal dimension (Table 1).

**Table 1:** Characteristics of our Chinese elderly participants.

Characteristics	Total (N = 50)	E/A ratio		p
		≤ 1.0 (n = 25)	> 1.0 (n = 25)	
Age, years	62.54 (11.74)	67.88 (6.90)	57.20 (13.20)	0.001
Sex, male	27(54.00%)	13 (52.00%)	14 (56.00%)	0.78
Ethnicity, Chinese	50 (100.00%)	25 (100.00%)	25 (100.00%)	–
**History of co-morbidity**				
Hypertension, yes	16 (32.00%)	11 (44.00%)	5 (20.00%)	0.13
Dyslipidemia, yes	20 (40.00%)	15 (60.00%)	5 (20.00%)	0.01
Diabetes, yes	4 (8.00%)	4 (16.00%)	0 (0.00%)	0.11
**Combination of co-morbidity**				
Without any condition	23 (46.00%)	6 (24.00%)	17 (68.00%)	0.004
With at least one condition	27 (54.00%)	19 (76.00%)	8 (32.00%)	
**Smoking status**				
Current smoker	34 (68.00%)	17 (68.00%)	17 (68.00%)	0.87
Past smoker	5 (10.00%)	3 (12.00%)	2 (8.00%)	
Never	11 (22.00%)	5 (20.00%)	6 (24.00%)	
BMI, kg/m^2^	23.93 (3.58)	24.62 (3.66)	23.23 (3.43)	0.17
Waist-to-hip ratio	0.87 (0.08)	0.89 (0.06)	0.84 (0.10)	0.02
**Blood pressure, mmHg**				
SBP	123.14 (17.99)	131.64 (17.81)	114.64 (13.88)	< 0.001
DBP	74.60 (10.08)	76.56 (9.98)	72.64 (10.00)	0.17
MAP	94.80 (11.24)	99.08 (11.45)	90.52 (9.43)	0.01
**Cardiac functional indices**				
LVEF (%)	72.96 (8.11)	74.09 (8.80)	71.83 (7.36)	0.33
MV E peak (m/s)	0.77 (0.19)	0.69 (0.11)	0.84 (0.22)	0.01
MV A peak (m/s)	0.71 (0.21)	0.86 (0.14)	0.56 (0.15)	< 0.001
E/A ratio	1.18 (0.49)	0.80 (0.09)	1.56 (0.43)	< 0.001
Septal S′ (m/s)	0.08 (0.02)	0.08 (0.02)	0.09 (0.02)	0.02
Septal E′ (m/s)	0.09 (0.03)	0.07 (0.02)	0.10 (0.02)	< 0.001
Septal A′ (m/s)	0.11 (0.02)	0.11 (0.02)	0.11 (0.02)	0.40
Lateral S′ (m/s)	0.11 (0.03)	0.10 (0.03)	0.12 (0.03)	0.03
Lateral E′ (m/s)	0.12 (0.03)	0.10 (0.02)	0.14 (0.03)	< 0.001
Lateral A′ (m/s)	0.12 (0.03)	0.12 (0.03)	0.12 (0.03)	0.46
E/E′ septal	9.36 (2.89)	9.92 (2.64)	8.81 (3.08)	0.18
E/E′ lateral	6.74 (2.09)	7.19 (2.09)	6.30 (2.04)	0.14
E/E′ average	7.77 (2.26)	8.24 (2.10)	7.30 (2.35)	0.14
**Retinal vascular parameters**				
CRAE (µm)	133.85 (11.51)	132.84 (13.05)	134.86 (9.89)	0.54
CRVE (µm)	199.14 (16.07)	194.83 (14.64)	201.46 (17.03)	0.15
DF-a (Df)	1.19 (0.08)	1.17 (0.08)	1.21 (0.08)	0.12
DF-v (Df)	1.21 (0.08)	1.18 (0.08)	1.23 (0.08)	0.02
CT-a (× 10^5^ unites)	5.45 (4.27)	5.65 (5.30)	5.29 (3.03)	0.77
CT-v (× 10^5^ unites)	7.79 (3.01)	7.39 (2.83)	8.19 (3.18)	0.35
BA-a (°)	69.74 (18.80)	68.22 (22.21)	71.27 (17.38)	0.60
BA-v (°)	76.62 (13.61)	75.60 (17.40)	77.64 (8.55)	0.60

Mean (SD) are presented for continuous variables and N (%) are presented for non-continuous variables.

*BMI* body mass index, *SBP* systolic blood pressure, *DBP* diastolic blood pressure, *MAP* mean arterial blood pressure, *LVEF* left ventricular ejection fraction, *MV E peak* peak velocity flow in early diastole, *MV A peak* peak velocity flow in late diastole by atrial contraction, *E/A ratio* peak velocity flow in early diastole/peak velocity flow in late diastole by atrial contraction, *Septal S*′ peak systolic septal mitral annular velocity, *Septal E*′ peak early diastolic septal mitral annular velocity, *Septal A*′ septal mitral annular velocity during atrial contraction, *Lateral S*′ peak systolic lateral annulus velocity, *Lateral E*′ peak early diastolic lateral annulus velocity, *Lateral A*′ lateral annulus velocity during atrial contraction, *E*/*E*′ *septal* ratio of mitral peak velocity flow in early diastole to peak early diastolic septal mitral annular velocity, *E*/*E*′ *lateral* ratio of mitral peak velocity flow in early diastole to peak early diastolic lateral annulus velocity, *E*/*E*′ *average* the ratio of MV E peak and average of Septal E′ and Lateral E′, *CRAE* central retinal arteriolar equivalent, *CRVE* central retinal venular equivalent, *DF-a* fractal dimension-arteriole, *DF-v* fractal dimension-venule, *CT-a* curvature tortuosity-arteriole, *CT-v* curvature tortuosity-venule, *BA-a* branching angle-arteriole, *BA-v* branching angle-venule.

**Figure 1:**
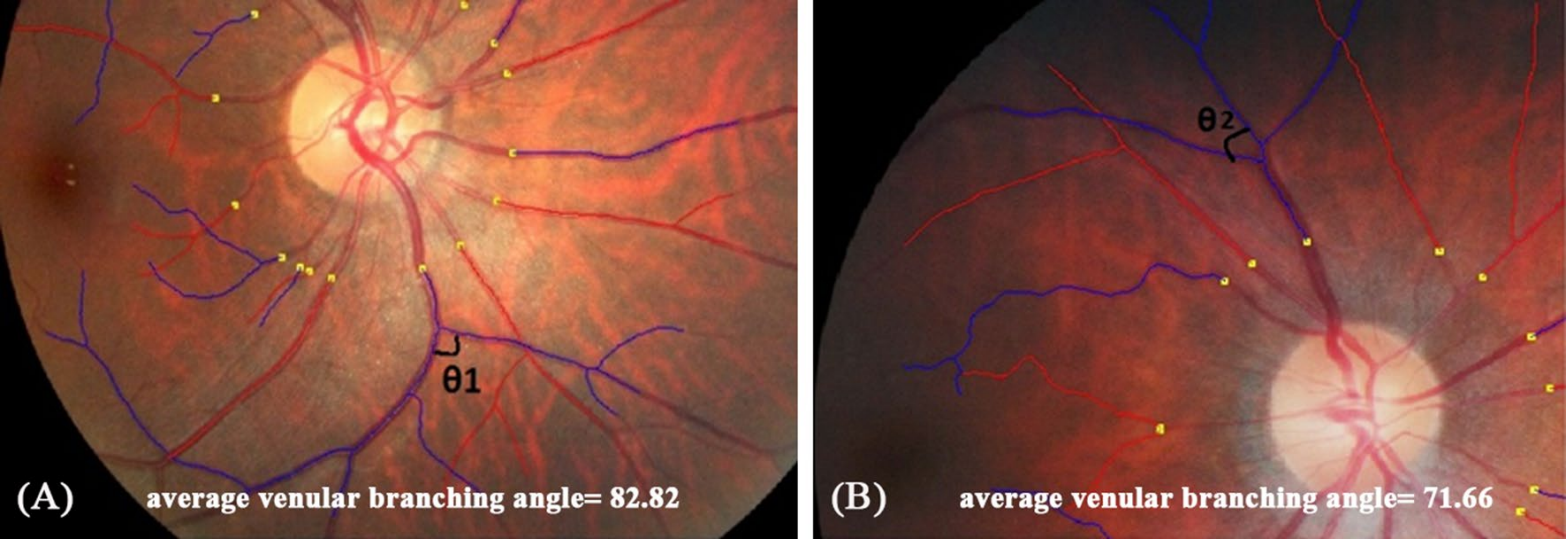
Examples of retinal fundus photographs from a participant with better left ventricular (LV) function (**A**) and a participant with poor LV function (**B**). Retinal venular branching angle is shown in images (**A**,**B**). Blue color indicates retinal venules, and red color indicates retinal arterioles. (**A**) showed average venular branching angle as 82.82°, while the participant was with better LV function (Septal S′ = 0.13 m/s, Lateral S′ = 0.16 m/s, and Lateral E′ = 0.19 m/s); (**B**) showed average venular branching angle as 71.66°, while the participant was with poor LV function (lower peak systolic septal mitral annular velocity (Septal S′) = 0.08 m/s, peak systolic lateral annulus velocity (Lateral S′) = 0.10 m/s, and peak early diastolic lateral annulus velocity (Lateral E′) = 0.09 m/s).

**Figure 2:**
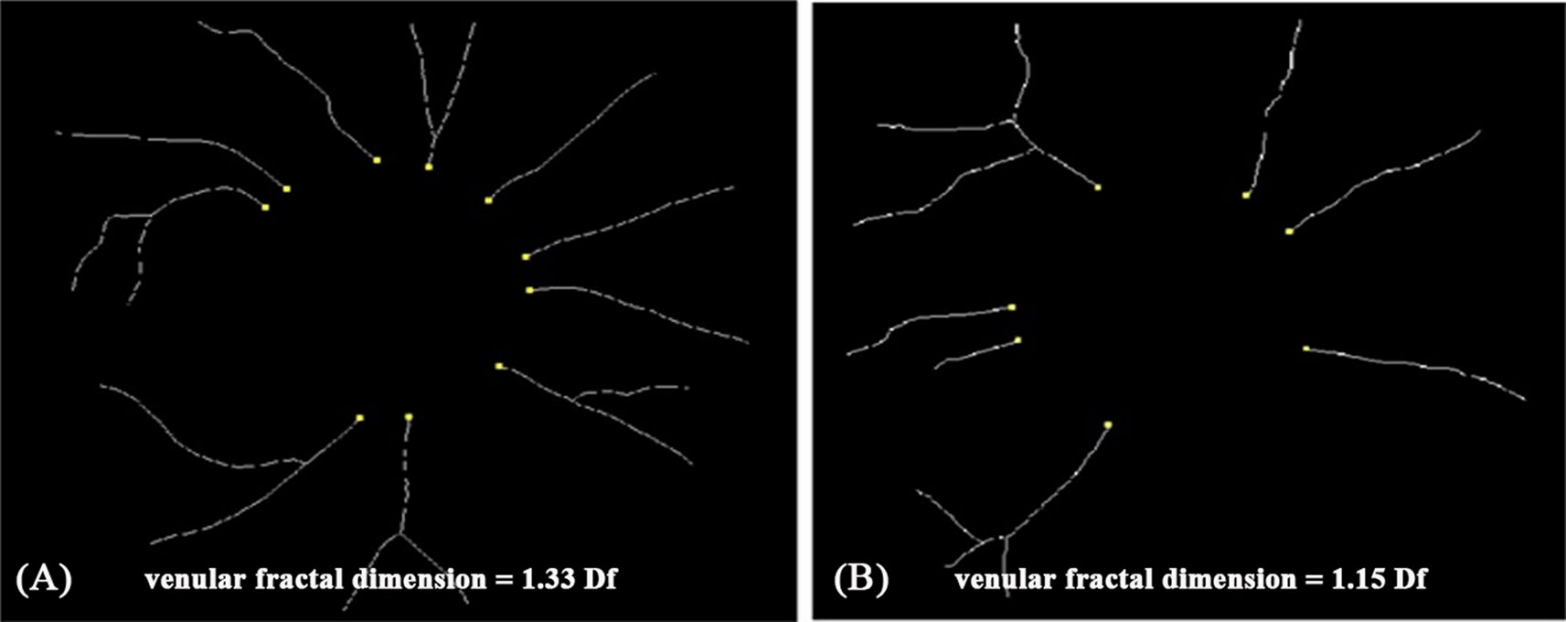
Examples of retinal fundus photographs from a participant with better left atrial (LA) function (**A**) and a participant with poor LA function (**B**). Retinal venular fractal dimension is shown in images (**A**,**B**). (**A**) showed retinal venular fractal dimension as 1.33 Df, while the participant was with better LA function (MV A peak = 0.49 m/s). (**B**) showed retinal venular fractal dimension as 1.15 Df, while the participants was with poor LA function (higher peak velocity flow in late diastole by atrial contraction (MV A peak) = 0.90 m/s).

After adjusting for covariates, each unit increase in MV A Peak that indicated poorer left atrium function was associated with lower retinal venular fractal dimension and higher retinal arteriolar curvature tortuosity, respectively (Table 2). Each unit increase in E/E′ lateral that indicated poorer left ventricular function was associated with smaller retinal venular branching angle (β: − 2.69°; 95% CI − 4.92, − 0.46) (Table 2). Furthermore, each unit decrease in other cardiac indices indicating poorer left ventricular function were consistently associated with smaller retinal venular branching angle (Septal S′: − 242.31°, − 475.53, − 9.09; Lateral S′: − 247.73°, − 376.14, − 119.32; Lateral E′: − 194.60°; − 351.68, − 37.52), accordingly (Table 2). Examples of retinal imaging showing microvascular geometry differences between patients with poorer and better cardiac function were shown in Figs. 1 and 2. However, no aforementioned cardiac functional indices were associated with retinal vascular calibres (Table S1).

**Table 2:** Associations between retinal vascular geometric parameters and cardiac functional indices.

Echo parameters	DF-a (Df) Beta (95% CI)	DF-v (Df) Beta (95% CI)	CT-a (× 10^5^ units) Beta (95% CI)	CT-v (× 10^5^ units) Beta (95% CI)	BA-a (°) Beta (95% CI)	BA-v (°) Beta (95% CI)
**Each unit increase: higher index indicating poorer cardiac function**						
*MV E peak*						
Model 1	− 0.01 (− 0.13, 0.11)	− 0.04 (− 0.15, 0.08)	4.80 (− 2.20, 11.81)	2.30 (− 2.67, 7.26)	− 3.27 (− 34.56, 28.01)	− 11.71 (− 33.58, 10.16)
Model 2	− 0.04 (− 0.18, 0.11)	− 0.03 (− 0.17, 0.10)	2.90 (− 5.43, 11.24)	1.10 (− 4.83, 7.02)	8.25 (− 28.60, 45.10)	− 3.05 (− 28.88, 22.78)
Model 3	− 0.03 (− 0.18, 0.11)	− 0.03 (− 0.17, 0.11)	2.06 (− 6.14, 10.27)	0.40 (− 5.36, 6.15)	4.20 (− 31.83, 40.24)	− 2.47 (− 28.75, 23.81)
*MV A peak*						
Model 1	− 0.06 (− 0.18, 0.06)	− 0.11 (− 0.22, 0.001)	**7.16 (0.17, 14.15)**	2.34 (− 2.74, 7.41)	− 10.00 (− 41.86, 21.87)	− 16.71 (− 38.80, 5.38)
Model 2	− 0.06 (− 0.19, 0.06)	− **0.12 (**− **0.24, -0.01)**	**9.28 (2.30, 16.26)**	3.40 (− 1.84, 8.63)	− 12.59 (− 45.55, 20.37)	− 21.17 (− 43.48, 1.15)
Model 3	− 0.06 (− 0.19, 0.08)	− **0.13 (**− **0.25, -0.004)**	**8.02 (0.76, 15.29)**	2.00 (− 3.33, 7.33)	− 23.64 (− 56.44, 9.17)	− 21.60 (− 45.19, 1.99)
*E/E′ septal*						
Model 1	− 0.003 (− 0.01, 0.01)	− 0.002 (− 0.01, 0.01)	0.20 (− 0.33, 0.73)	0.01 (− 0.37, 0.38)	0.98 (− 1.34, 3.30)	− 0.63 (− 2.28, 1.01)
Model 2	− 0.003 (− 0.01, 0.01)	− 0.002 (− 0.01, 0.01)	0.22 (− 0.31, 0.76)	0.01 (− 0.37, 0.40)	0.71 (− 1.67, 3.08)	− 0.88 (− 2.53, 0.76)
Model 3	− 0.002 (− 0.01, 0.01)	− 0.002 (− 0.01, 0.01)	0.13 (− 0.41, 0.67)	− 0.07 (− 0.45, 0.31)	0.26 (− 2.10, 2.61)	− 0.85 (− 2.55, 0.85)
*E/E′ lateral*						
Model 1	− 0.002 (− 0.02, 0.01)	− 0.004 (− 0.02, 0.01)	0.40 (− 0.34, 1.14)	− 0.04 (− 0.56, 0.48)	0.04 (− 3.23, 3.31)	− **2.92 (**− **5.06, **− **0.78)**
Model 2	− 0.003 (− 0.02, 0.01)	− 0.004 (− 0.02, 0.01)	0.32 (− 0.43, 1.06)	− 0.10 (− 0.63, 0.44)	0.32 (− 3.00, 6.64)	− **2.70 (**− **4.87, **− **0.52)**
Model 3	− 0.002 (− 0.02, 0.01)	− 0.004 (− 0.02, 0.01)	0.21 (− 0.53, 0.95)	− 0.19 (− 0.71, 0.33)	− 0.20 (− 3.46, 3.06)	− **2.69 (**− **4.92, **− **0.46)**
*E/E′ average*						
Model 1	− 0.002 (− 0.01, 0.01)	− 0.004 (− 0.02, 0.01)	0.34 (− 0.36, 1.04)	− 0.05 (− 0.54, 0.44)	0.63 (− 2.45, 3.71)	− **2.15 (**− **1.24, **− **0.06)**
Model 2	− 0.003 (− 0.02, 0.01)	− 0.003 (− 0.02, 0.01)	0.29 (− 0.41, 0.99)	− 0.08 (− 0.58, 0.42)	0.65 (− 2.45, 3.76)	− **2.09 (**− **4.17, **− **0.01)**
Model 3	− 0.002 (− 0.01, 0.01)	− 0.003 (− 0.02, 0.01)	0.18 (− 0.52, 0.88)	− 0.18 (− 0.67, 0.30)	0.09 (− 2.98, 3.16)	− 2.08 (− 4.23, 0.06)
**Each unit decrease: lower index indicating poorer cardiac function**						
*LVEF*						
Model 1	0.001 (− 0.002, 0.004)	0.001 (− 0.001, 0.004)	− 0.12 (− 0.30, 0.05)	− 0.05 (− 0.17, 0.07)	0.56 (− 0.19, 1.32)	0.39 (− 0.14, 0.93)
Model 2	0.001 (− 0.002, 0.004)	0.001 (− 0.001, 0.004)	− 0.11 (− 0.28, 0.07)	− 0.04 (− 0.17, 0.09)	0.51 (− 0.26, 1.28)	0.33 (− 0.21, 0.87)
Model 3	0.001 (− 0.002, 0.004)	0.001 (− 0.001, 0.004)	− 0.12 (− 0.29, 0.05)	− 0.05 (− 0.17, 0.07)	0.46 (− 0.29, 1.21)	0.34 (− 0.20, 0.89)
*E/A ratio*						
Model 1	− 0.02 (− 0.07, 0.03)	− 0.02 (− 0.07, 0.02)	0.74 (− 2.24, 3.72)	0.17 (− 1.92, 2.27)	− 2.93 (− 15.99, 10.13)	− 1.01 (− 10.27, 8.24)
Model 2	− 0.01 (− 0.07, 0.05)	− 0.04 (− 0.09, 0.02)	2.60 (− 0.78, 5.98)	1.12 (− 1.31, 3.56)	− 8.33 (− 23.40, 6.74)	− 6.26 (− 16.78, 4.26)
Model 3	− 0.01(− 0.07, 0.05)	− 0.04 (− 0.09, 0.02)	2.24 (− 1.10, 5.58)	0.81 (− 1.56, 3.19)	− 10.38 (− 24.97, 4.21)	− 6.08 (− 16.81, 4.65)
*Septal S′*						
Model 1	− 0.94 (− 2.15, 0.27)	− 0.61 (− 1.78, 0.57)	34.21 (− 39.86, 108.28)	12.80 (− 39.45, 65.05)	− 115.45 (− 441.07, 210.18)	− 154.83 (− 381.87,72.21)
Model 2	− 0.95 (− 2.24, 0.35)	− 0.75 (− 2.00, 0.51)	56.08 (− 20.21, 132.38)	24.31 (− 30.58, 79.19)	− 188.88 (− 528.52, 150.77)	− **234.66 (**− **464.93,-4.39)**
Model 3	− 0.99 (− 2.30, 0.33)	− 0.78 (− 2.05, 0.49)	63.90 (− 10.16, 137.96)	30.42 (− 22.39, 83.22)	− 156.92 (− 489.16, 175.32)	− **242.31 (**− **475.53, **− **9.09)**
*Septal E′*						
Model 1	− 0.23 (− 1.24, 0.78)	− 0.01 (− 0.98, 0.96)	− 7.66 (− 68.86, 53.54)	− 10.88 (− 53.67, 31.92)	65.15 (− 202.38, 332.69)	− 13.38 (− 203.10, 176.34)
Model 2	− 0.12 (− 1.40, 1.16)	− 0.17 (− 1.40, 1.06)	15.68 (− 59.47, 90.83)	− 1.36 (− 54.70, 51.98)	− 66.52 (− 397.70, 264.66)	− 165.02 (− 391.73, 61.70)
Model 3	− 0.12 (− 1.41, 1.17)	− 0.17 (− 1.41, 1.07)	16.66 (− 56.58, 89.92)	− 0.57 (− 51.95, 50.82)	− 61.93 (− 383.06, 259.21)	− 165.75 (− 394.75, 63.25)
*Septal A′*						
Model 1	− 0.13 (− 1.46, 1.20)	0.51 (− 0.76, 1.78)	− 20.98 (− 101.04, 59.07)	− 28.43 (− 84.06, 27.2)	151.82 (− 196.96, 500.59)	− 34.33 (− 282.92, 214.26)
Model 2	− 0.09 (− 1.46, 1.28)	0.50 (− 0.81, 1.80)	− 19.92 (− 100.25, 60.41)	− 28.11 (− 84.53, 28.32)	123.40 (− 229.55, 476.36)	− 57.25 (− 305.03, 190.53)
Model 3	− 0.15 (− 1.55, 1.26)	0.47 (− 0.88, 1.81)	− 7.11 (− 86.97, 72.75)	− 18.13 (− 73.76, 37.49)	191.31 (− 153.72, 536.34)	− 68.83 (− 323.20, 185.55)
*Lateral S′*						
Model 1	0.02 (− 0.77, 0.82)	− 0.10 (− 0.86, 0.66)	12.14 (− 35.67, 59.96)	− 15.22 (− 48.52, 18.08)	21.77 (− 188.15, 231.68)	− **234.20 (**− **365.51, **− **102.89)**
Model 2	0.04 (− 0.78, 0.85)	− 0.12 (− 0.90, 0.67)	15.67 (− 32.02, 63.37)	− 13.47 (− 47.17, 20.24)	11.20 (− 199.97, 222.36)	− **247.80 (**− **374.98, **− **120.62)**
Model 3	0.04 (− 0.79, 0.86)	− 0.12 (− 0.90, 0.67)	15.58 (− 30.91, 62.06)	− 13.55 (− 45.98, 18.89)	10.74 (− 193.98, 215.45)	− **247.73 (**− **376.14, **− **119.32)**
*Lateral E′*						
Model 1	0.09 (− 0.77, 0.95)	0.02 (− 0.80, 0.84)	− 8.07 (− 59.87, 43.73)	− 22.81 (− 58.51, 12.89)	− 26.15 (− 200.81, 253.12)	− 141.12 (− 296.23, 13.99)
Model 2	0.15 (− 0.77, 1.07)	− 0.03 (− 0.91, 0.85)	3.41 (− 50.53, 57.36)	− 18.15 (− 55.97, 19.67)	− 9.66 (− 247.37, 228.06)	− **195.04 (**− **350.50, **− **39.59)**
Model 3	0.15 (− 0.77, 1.71)	− 0.03 (− 0.92, 0.86)	2.73 (− 49.87, 55.34)	− 18.71 (− 55.07, 17.66)	− 12.87 (− 243.33, 217.59)	− **194.60 (**− **351.68, **− **37.52)**
*Lateral A′*						
Model 1	0.39 (− 0.33, 1.10)	0.65 (− 0.02, 1.31)	− 25.23 (− 68.32, 17.86)	− 25.22(− 54.94, 4.50)	72.68 (− 117.64, 263.00)	− 66.34 (− 200.41, 67.73)
Model 2	0.41 (− 0.32, 1.14)	0.64 (− 0.05, 1.33)	− 23.12 (− 66.30, 20.05)	− 24.16 (− 54.27, 5.94)	59.95 (− 131.93, 251.82)	− 78.98 (− 211.71, 53.75)
Model 3	0.40 (− 0.35, 1.14)	0.63 (− 0.07, 1.33)	− 19.00 (− 61.52, 23.53)	− 20.88 (− 50.23, 8.46)	81.59 (− 104.78, 267.96)	− 83.14 (− 217.88, 51.59)

Model 1 adjusted for age and sex.

Model 2 adjusted for age, sex, MAP and BMI.

Model 3 adjusted for age, sex, MAP, BMI and combination of co-morbidity.

Beta and 95% CI are presented in the table, and values with statistically significance (p < 0.05) are highlighted in boldface font.

*MV E Peak* peak velocity flow in early diastole, *MV A peak* peak velocity flow in late diastole by atrial contraction, *E/E*′ *septal* ratio of mitral peak velocity flow in early diastole to peak early diastolic septal mitral annular velocity, *E/E*′ lateral: ratio of mitral peak velocity flow in early diastole to peak early diastolic lateral annulus velocity, *E/E*′ average: the ratio of MV E Peak and average of Septal E′ and Lateral E′, *LVEF* left ventricular ejection fraction, *E/A ratio* peak velocity flow in early diastole/peak velocity flow in late diastole by atrial contraction, *Septal S*′ peak systolic septal mitral annular velocity, *Septal E*′ peak early diastolic septal mitral annular velocity, *Septal A*′ septal mitral annular velocity during atrial contraction, *Lateral S*′ peak systolic lateral annulus velocity, *Lateral E*′ peak early diastolic lateral annulus velocity, *Lateral A*′ lateral annulus velocity during atrial contraction, *DF-a* fractal dimension-arteriole, *DF-v* fractal dimension-venule, *CT-a* curvature tortuosity-arteriole, *CT-v* curvature tortuosity-venule, *BA-a* branching angle-arteriole, *BA-v* branching angle-venule.

## Discussion

Our study observed significant associations between subclinical changes in left ventricular function (i.e. Septal S′, Lateral S′, Lateral E′ and E/E′ lateral) and left atrial function (i.e. MV A Peak) with suboptimal retinal venular geometry (i.e. lower fractal dimension and smaller branching angle), among Chinese subjects without prior history of CVD. Our findings suggested that suboptimal retinal venular geometry could be a potential proxy to mirror cardiac dysfunction.

It is well-known that pathogenic risk factors such as chronic stress and systemic inflammation can lead to adverse alterations in cardiac structure and function (i.e. enlarged volume and reduced cardiac output)^5,6^, which can ultimately progress to CVD such as HF^7^. However, whether there is a parallel microcirculatory disturbance along with the cardiac changes in structure and function has yet to be investigated via conventional techniques. Retinal microvasculature carries substantial information on the general microcirculation. For example, vessel rarefaction and collapse—leading to reduction in vascular fractal dimension—is associated with hypoxia^8^, and increased vessel tortuosity is indicative of vessel wall dysfunction and blood–retina barrier damage^9^, and narrowing venular branching angle is indicative of increased levels of oxidative stress^10^. Our observation suggested that the changes in retina vessels (i.e., arteriolar fractal dimension and venular branching angle reduction) may reflect increased systematic endothelial dysfunction and oxidative stress, resulting in changes in cardiac function. Intriguingly, we found that venular changes, instead of arteriolar changes in the retina, were associated with poorer cardiac function. Our previous investigations have demonstrated that for cardiac structure, changes in retinal arterioles were more sensitive to subclinical alterations than retinal venules^11^. These differential associations may suggest adaptive responses occurring within venules, predating more gross changes within cardiac structure that are reflected later within the retinal arterioles^12^.

Our exploratory study has identified novel associations between cardiac function and retinal venular geometry, capitalizing on detailed cardiovascular and retinal measurements. Nevertheless, we acknowledged the limitations of our study. Firstly, the small sample in this study might have restricted our power to detect more associations with significance. Secondly, while we did not correct our findings for multiple comparisons, we took guidance from other studies that have also similar pre-hypothesized independent variables in their statistical associations^13^. Thirdly, we acknowledged that other variables such as alcohol consumption, socio-economic status, medications, plasma glycosylated hemoglobin (HbA1c) and diabetes duration, which were not available for this study sample, may contribute to residual confounding. Fourthly, this study involved Chinese subjects in an Asian setting for which observed findings might not be generalizable to other ethnic populations. Finally, the cross-sectional nature of our study design precludes inferences about causality. Future larger studies, in other ethnic cohorts, extended over time, may be necessary to verify our findings.

In conclusion, our findings showed that adverse changes in cardiac function might be mirrored by suboptimal retinal vascular geometry. Further longitudinal studies with larger samples with right ventricular and atrial measures are warranted to not only confirm our findings, but also provide more evidence on the underlying pathophysiology for CVD and even HF development.

## Materials and methods

This study conducted a cross-sectional analysis between cardiac function and retinal microvascular geometry among Chinese (Han ethnicity) subjects (n = 50), who were recruited from the Cardiac Ageing Study (CAS)^14^ and also underwent retinal examination. Briefly, CAS is a prospective study initiated in 2014 that examines characteristics and determinants of cardiovascular function of community adults aged 38 years and above. CAS excluded subjects who had self-reported history of physician-diagnosed cardiovascular disease (such as coronary heart disease, atrial fibrillation and stroke) or cancer, and required all participants to complete a standardized questionnaire collecting personal medical history and coronary risk variables. Such details have been described in prior publications^14^. The study complied with the Declaration of Helsinki. All participants agreed and signed the written informed consent upon enrolment. The SingHealth Centralised Institutional Review Board (2014/628/C) approved the study protocol.

Transthoracic echocardiography (ALOKA α10 with a 3.5 MHz probe, Hitachi Medical, Wallington, CT, USA) included 2-D, M-mode, pulse Doppler and tissue Doppler imaging was performed to evaluate the cardiac function, namely Peak systolic septal mitral annular velocity (Septal S′), Peak systolic lateral annulus velocity (Lateral S′), Peak early diastolic lateral annulus velocity (Lateral E′), peak velocity flow in early diastole E (MV E peak), peak velocity flow in late diastole by atrial contraction (MV A peak) and their ratio (E/A ratio), and the ratio of MV E peak to Lateral E′ (E/E′ lateral), according to the guidelines of American Society of Echocardiography^15^. At the end of each examination, all measurements among three cardiac cycles were averaged and adjusted with the interbeat interval by the same echocardiographer. Retinal vascular imaging was performed (Canon CR-1, 40D SLR digital retinal camera backing, Canon Inc., Tokyo, Japan) and accessed (Singapore I Vessel Assessment (SIVA) version 3.0, Singapore Eye Research Institute, Singapore) to obtain retinal vascular parameters including calibre, branching angle, curvature tortuosity and fractal dimension, according to a standard protocol described elsewhere^16^. The same grader reanalyzed the vessels in 10% of the total retinal images, and intragrader correlation coefficient is consistently > 80% across all retinal vascular geometric indices.

Covariates including age, sex, mean arteriolar pressure (MAP), body mass index (BMI, calculated as weight in kilograms divided by the square of height in meters) and history of comorbidity (i.e. dyslipidaemia, hypertension, diabetes mellitus) were collected during clinical visit interview.

Mean and standard deviation (SD) and counts and percentages were used to describe all variables. Characteristics of groups according to E/A ratios (> 1.0 vs. ≤ 1.0; E/A ratio of ≤ 1.0 is the reflective of impaired myocardial relaxation^17^) were compared. Multiple linear regressions were conducted to assess the associations between cardiac function and retinal vascular measures. Three models were applied: Model 1, adjusted for age and sex; Model 2, Model 1 and additionally adjusting for MAP and BMI; Model 3, Model 2 and additionally adjusting for history of comorbidity. For all analyses, we defined a significant p-value (two-tailed) as 0.05. We performed all statistical analyses using IBM SPSS software version 23.0 (SPSS, IBM, Chicago, USA).

## Data availability

The datasets generated during and/or analysed during the current study are not publicly available due to privacy protection but are available from the corresponding author on reasonable request.

## Supplementary information

Supplementary Table.
