# Supplementary material for: Associations between cardiac function and retinal microvascular geometry among Chinese adults
Source: Sci Rep. 2020 Sep 9;10:14797. doi: 10.1038/s41598-020-71385-0 (PMC7481218; doi:10.1038/s41598-020-71385-0)
Supplement: Supplementary file 1 — Supplementary Table. [file 41598_2020_71385_MOESM1_ESM.docx]

**Associations between Cardiac Function and Retinal Microvascular Geometry among Chinese Adults**

Lihua Huang ^1^, Wei-Qing Chen ^1^, Izzuddin M. Aris^2^, Louis L.Y. Teo ^3, 4^, Tien Yin Wong ^5, 6, 7^, Angela S. Koh ^3, 4, *^, Ling-Jun Li ^3, 5, 8, *^

Supplementary Table 1 Associations between Retinal Vascular Calibers and Cardiac Functional Indices.

| Each unit increase in cardiac functional indices | CRAE (µm)  Beta  (95%CI) | | |  | CRVE (µm)  Beta  (95%CI) | | |
| --- | --- | --- | --- | --- | --- | --- | --- |
|  | Model 1 | Model 2 | Model 3 |  | Model 1 | Model 2 | Model 3 |
| LVEF | 0.10  (-0.37,0.56) | 0.08  (-0.36, 0.52) | 0.09  (-0.36, 0.53) |  | 0.40  (-0.21, 1.01) | 0.44  (-0.14, 1.01) | 0.44  (-0.15, 1.02) |
| MV E Peak | 4.49  (-14.26,23.25) | 0.09  (-20.75, 20.93) | -0.71  (-21.85, 20.43) |  | -12.94  (-37.73,11.85) | -15.31  (-42.55, 11.93) | -15.25  (-43.01, 12.51) |
| MV A Peak | 0.11  (-19.12,19.33) | 5.93  (-12.71, 24.56) | 4.56  (-15.09, 14.21) |  | -3.80  (-29.42, 21.82) | 0.66  (-24.17, 25.48) | 1.37  (-24.86, 27.61) |
| E/A ratio | 1.59  (-6.26, 9.43) | -1.41  (-10.02, 7.20) | -1.06  (-9.81, 7.69) |  | -2.30  (-12.76, 8.17) | -3.75  (-15.12, 7.63) | -3.94  (-15.54, 7.66) |
| Septal S’ | -42.19 (-238.46, 154.08) | -77.15 (-270.07, 115.77) | -84.53 (-279.62, 110.56) |  | -7.82 (-270.20, 254.57) | 3.09 (-254.62, 260.80) | 5.37 (-256.80, 267.54) |
| Septal E’ | -10.30 (-171.38, 150.79) | 2.24 (-181.95, 192.43) | 4.35 (-184.23, 192.94) |  | -81.80 (-295.38, 131.79) | 14.85 (-233.35, 263.05) | 15.16 (-236.02, 266.34) |
| Septal A’ | 34.99 (-175.98, 245.97) | 56.78 (-142.73, 256.29) | 46.50 (-158.20, 251.19) |  | -90.00 (-370.56, 190.56) | -47.42 (-312.58, 217.75) | -44.85 (-317.84, 228.13) |
| Lateral S’ | -58.42  (-183.37, 66.52) | -65.28  (-182.77, 52.21) | -65.19  (-183.53, 53.15) |  | -117.06  (-281.74, 47.61) | -117.09  (-271.04, 36.87) | -117.12 (-272.89, 38.66) |
| Lateral E’ | 9.97 (-126.40, 146.35) | -12.02 (-146.10, 122.06) | -11.40 (-146.48, 123.67) |  | -90.75 (-270.75, 89.24) | -95.98 (-271.44, 79.47) | -96.22 (-273.76, 81.33) |
| Lateral A’ | 45.32  (-68.94, 159.58) | 46.81  (-60.99, 154.62) | 43.40  (-66.03, 152.83) |  | -55.17  (-207.80, 97.45) | -45.11  (-188.66, 98.43) | -44.32 (-190.54, 101.91) |
| E/E’ septal | 0.23  (-1.17, 1.63) | 0.02  (-1.33, 1.36) | -0.07  (-1.46, 1.31) |  | -0.35  (-2.22, 1.51) | -0.82  (-2.59, 0.94) | -0.83  (-2.65, 1.00) |
| E/E’ lateral | 0.38  (-1.58, 2.34) | 0.19  (-1.68, 2.06) | 0.09  (-1.82, 2.00) |  | 0.34  (-2.28, 2.95) | 0.34  (-2.15, 2.82) | 0.38  (-2.17, 2.92) |
| E/E’ average | 0.45  (-1.40, 2.30) | 0.22  (-1.54, 1.97) | 0.11  (-1.69, 1.91) |  | 0.03  (-2.45, 2.50) | -0.21  (-2.53, 2.12) | -0.18  (-2.57, 2.22) |

Abbreviations: CRAE: Central retinal arteriolar equivalent; CRVE: Central retinal venular equivalent; LVEF: left ventricular ejection fraction; MV E Peak: Peak velocity flow in early diastole; MV A Peak: Peak velocity flow in late diastole by atrial contraction; E/A ratio: Peak velocity flow in early diastole /Peak velocity flow in late diastole by atrial contraction; Septal S’: Peak systolic septal mitral annular velocity; Septal E’: Peak early diastolic septal mitral annular velocity; Septal A’: Septal mitral annular velocity during atrial contraction; Lateral S’: Peak systolic lateral annulus velocity; Lateral E’: Peak early diastolic lateral annulus velocity; Lateral A’: Lateral annulus velocity during atrial contraction; E/E’ septal: ratio of mitral peak velocity flow in early diastole to peak early diastolic septal mitral annular velocity; E/E’ lateral: ratio of mitral peak velocity flow in early diastole to peak early diastolic lateral annulus velocity; E/E’ average: the ratio of MV E Peak and average of Septal E’ and Lateral E’.

*Beta and 95%CI are presented in the table.

Model 1 adjusted for sex and age

Model 2 adjusted for sex, age, MAP and BMI

Model 3 adjusted for sex, age, MAP, BMI and combination of co-morbidity
